# High-Throughput Sequencing Reveals Single Nucleotide Variants in Longer-Kernel Bread Wheat

**DOI:** 10.3389/fpls.2016.01193

**Published:** 2016-08-08

**Authors:** Feng Chen, Zibo Zhu, Xiaobian Zhou, Yan Yan, Zhongdong Dong, Dangqun Cui

**Affiliations:** National Key Laboratory of Wheat and Maize Crop, Collaborative Innovation Center of Henan Grain Crops, Agronomy College, Henan Agricultural UniversityZhengzhou, China

**Keywords:** wheat, ethyl methanesulfonate (EMS), mutation, next-generation sequencing, transcriptome, single nucleotide variations (SNVs)

## Abstract

The transcriptomes of bread wheat Yunong 201 and its ethyl methanesulfonate derivative Yunong 3114 were obtained by next-sequencing technology. Single nucleotide variants (SNVs) in the wheat strains were explored and compared. A total of 5907 and 6287 non-synonymous SNVs were acquired for Yunong 201 and 3114, respectively. A total of 4021 genes with SNVs were obtained. The genes that underwent non-synonymous SNVs were significantly involved in ATP binding, protein phosphorylation, and cellular protein metabolic process. The heat map analysis also indicated that most of these mutant genes were significantly differentially expressed at different developmental stages. The SNVs in these genes possibly contribute to the longer kernel length of Yunong 3114. Our data provide useful information on wheat transcriptome for future studies on wheat functional genomics. This study could also help in illustrating the gene functions of the non-synonymous SNVs of Yunong 201 and 3114.

## Introduction

Wheat (*Triticum aestivum* L.) is one of the three most important cereals (i.e., maize, rice, and wheat), with more than 600 million tons harvested annually (Shewry, 2009). In wheat breeding programs, yield can be divided into three components, namely, spike number per acre, grain number per spike, and thousand-grain weight. Grain weight is mainly determined by grain size in bread wheat. Therefore, kernel size, which is extensively studied in wheat breeding programs, is a key factor affecting wheat yield. Grain size is mainly controlled by heredity. Previously, many genes related to grain size of rice have been successfully cloned (Song et al., 2007; Wang et al., 2008, 2012; Weng et al., 2008; Li et al., 2011). Some quantitative trait loci (QTLs) controlling grain shape and size in bread wheat have been mapped through marker analysis in different wheat populations (Breseghello and Sorrells, 2006; Wu et al., 2015). Many of them were associated with the orthologs of rice grain traits QTLs (Lu et al., 2015; Wu et al., 2015; Zanke et al., 2015). For example, *GRAIN SIZE 3* (GS3) is a major QTL for rice grain length and weight (Fan et al., 2006), *TaGS-D1* was the syntenic gene in wheat (Zhang et al., 2014). These studies revealed that grain size of bread wheat is regulated via a complex molecular genetic mechanism. However, in bread wheat, the genetic study of grain size has been limited to date because of its large *de novo* genome. Next-generation sequencing (NGS) technology (Goff et al., 2002) provides a novel method to identify, map, and quantify transcriptomes (Kyndt et al., 2012); this method can also be used for rapid characterization of transcript sequences, gene expression (Wang et al., 2009), and genomic variation in polyploid plants with *de novo* genomes (Goff et al., 2002; Marioni et al., 2008). The first homolog-specific sequence assembly of wheat transcriptome is based on Roche 454 and Illumina GAIIx (Schreiber et al., 2012). Moreover, the genome of wheat and of its relative were analyzed by recent studies utilizing high-throughput sequencing, which provided references for further study (Brenchley et al., 2012; Ling et al., 2013; The International Wheat Genome Sequencing Consortium [IWGSC], 2014). Subsequently, large amount of advances have been acquired on wheat transcriptome (Duan et al., 2012; Pfeifer et al., 2014; Tanaka et al., 2014; Pingault et al., 2015).

The correlation between the genetic variants and the phenotypes is still a central question for crop improvements. Ethyl methanesulfonate (EMS) induces a large spectrum of mutations, including truncations and missense mutations, thereby allowing to be a readily used chemical in traditional breeding programs because of its flexibility, non-transgenicity, and stable inheritability (McCallum et al., 2000; Henikoff et al., 2004). Furthermore, EMS can create random point mutations at high density in polyploid plants. Many studies has focused on creation of mutations via EMS in specific genes of plants, and all the mutations are G–A or C–T transitions in bread wheat (Feiz et al., 2009; Uauy et al., 2009; Slade et al., 2012; Wang et al., 2014). Through RNAseq, large number of genetic variants across the transcriptomes could be identified. The possible transcriptional mechanism of trait regulation due to genetic variants including single nucleotide polymorphism substitutions has been reported in many crops such as in rice (Mao et al., 2010), but still insufficient in wheat.

A Chinese wheat cultivar Yunong 201, which was released in 2006 (No. Yushenmai2006006), is a high-quality noodle wheat strain that is disease-resistant. Meanwhile, its EMS mutagenesis-derived Yunong 3114 shows longer kernel length and higher production. In the present study, to better understand the genetic basis of kernel size in bread wheat, Illumina (Solexa) sequencing technology was applied in bread wheat Yunong 201 and its EMS mutant line Yunong 3114 to generate their transcriptomes. Gene profiles of Yunong 201 and 3114 were obtained by *de novo* sequencing. Single nucleotide variants (SNVs) were analyzed. This study provided important information to further understand the transcriptome of hexaploid wheat and determine the wheat-specific genes related to grain size.

## Materials and Methods

### Plant Materials

A Chinese winter wheat cultivar Yunong 201 showing outstanding dry, white noodle quality was treated by 1.0% EMS (0.1 mol/L Na_2_HPO_4_ ⋅12H_2_O, pH 7.0). An elite M_2_ line was screened from the EMS-mutagenized population encompassing 2000 lines because of its longer kernel length and higher grain weight; this line was self-crossed for three times into a M_5_ line Yunong 3114. Yunong 201 and its derived line Yunong 3114 were planted and grown at the Zhengzhou Scientific Research and Education Center of Henan Agricultural University during 2011–2012 cropping seasons under non-stressed conditions. Grains were collected at different developmental stages from both lines at 7, 14, 21, 28, and 35 days after flowering, and mature seeds (stored 1 year at 4°C after harvested); pooled samples were obtained by mixing eighteen grains from three spikes of each wheat strain at each developmental stage mentioned above; these samples were used for the generation of small RNA libraries of Yunong 201 and 3114.

### RNA Sequencing

The total RNA of each sample was extracted with Trizol Reagent (Invitrogen), according to the manufacturer’s instructions. Full-length cDNAs were synthesized with a TruSeq RNA Sample Preparation Kit (Illumina), according to the manufacturer’s protocol. The cDNA libraries were sequenced on Illumina Hiseq 2000, according to the manufacturer’s instructions. A 10 Gb raw reads were obtained from each cDNA library, and a total of 120 Gb raw reads were generated from the different developmental staged seeds of Yunong 201 and 3114.

### Pretreatment of Data and Development of Unigenes

Raw reads were first trimmed by using SeqClean^1^ 86–64, Newbler 2.5.3 (Kumar and Blaxter, 2010), and Lucy 1.20p (Chou and Holmes, 2001) to remove low-quality reads, vector sequences, and reads whose length was less than 50 bp. The trimmed reads were subjected to polygenetic analysis with CD-HIT version 4.0 (Huang et al., 2010) at a sequence identity threshold of 99%. Possible contaminated sequences were removed by aligning to the NT library. The trimmed non-redundant reads were assembled to contigs by Newbler 2.5.3. One or more contigs were assembled to isotigs. A total of 12 groups of isotigs were developed from the small RNA libraries of Yunong 201 and 3114.

### Discovery of SNV

All SNVs of Yunong 201 and 3114 were obtained based on the wheat reference sequence mapping (URGI)^2^ (He et al., 2014). We focused on the sites with non-synonymous mutation. EMS treatment can trigger high-density point mutation on wheat material. We selected the mutation sites occurring in all samples. To explore the different biological mechanisms between Yunong 201 and 3114, three SNV types were significant. The first SNV type was that the mutation sites were same between Yunong 201 and 3114, and the mutation was non-synonymous as compared with reference sequence, but the nucleotides after mutation were different between Yunong 201 and 3114, we considered this mutation significant. The second type was that the nucleotides of Yunong 201 were changed into other nucleotides, but the nucleotides of Yunong 3114 were still the same as the reference sequence. The third SNV indicated that mutation occurred specifically in Yunong 3114, but not in Yunong 201. Thus, we studied the same and different mutation sites between Yunong 201 and Yunong 3114.

### Sequence Annotation

Isotigs and singlets sequences were subjected to polygenetic analysis by CD-HIT version 4.0 (Huang et al., 2010) with an identity of 95%. Chromosome mapping and comparison with known genome segments were also performed. Using the BLAST program (Camacho et al., 2009) (a searched threshold of 1e–10), we searched for unigenes in EST libraries of 10 species related to NCBI^3^ and DFCI^4^. Nucleotides and protein were separately annotated by comparing the unigenes with those in the NT library (*E*-value < 1e–5) and the NR library (BLASTX; *E*-value < 1e–5; similarity of protein >30%). Moreover, we also annotated the sequences based on the wheat sequence database of French National Institute for Agricultural Research^5^ (INRA). We mainly referenced the sequence of *T*. *aestivum* chromosome 3B (ta3b, genomic scaffold, cultivar Chinese Spring) in URGI.

### Functional Annotation

We identified the annotated unigene sequences for the possible functions through the Gene Ontology Consortium^6^. Sequences were mapped to the reference authoritative pathways in Kyoto Encyclopedia of Genes and Genomes (KEGG)^7^ to determine the active biological pathways in annotated unigene sequences.

### Heat Map Analysis

Heat map is shown for preliminary SNV gene expression (logFoldChange) in the mutant strain at different developmental stages with respect to the wild type. Heat map was generated via Microsoft Excel, and logFold-Change values were used. The highest, medium, and lowest values are shown in color red, black, and green, respectively.

## Results

### Investigation of Agronomic Traits in Yunong 201 and 3114

**FIGURE 1:**
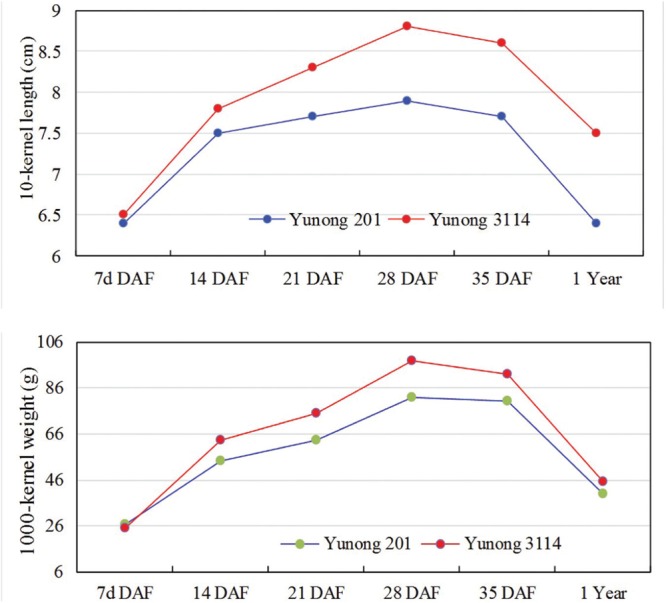
**Kernel size parameters of Yunong 201 and 3114 during six seed developmental stages.** DAF, days after flowering.

Wheat strain Yunong 3114 showed longer kernel length, higher grain weights, and higher yield compared with Yunong 201 (data not shown); the former did not also show obvious difference on kernel width (detailed data in **Figure 1**). Therefore, the higher grain weight of Yunong 3114 resulted from its relatively longer kernel length than that of Yunong 201. The grains with different developmental stages at 7, 14, 21, 28, and 35 days after flowering, and mature seeds (stored 1 year at 4°C after harvested) were collected from both lines; these grains were used to generate six pairs of small RNA libraries of Yunong 201 and 3114 for sequencing. Transcriptomes of both Yunong 201 and 3114 were obtained for the SNVs analysis.

### Discovery of SNVs in Yunong 201 and 3114

**FIGURE 2:**
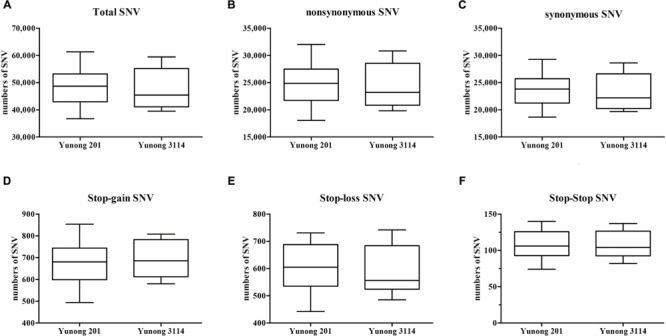
**Single nucleotide variants (SNVs) between Yunong 201 and 3114. (A–C)** The total SNV, non-synonymous, and synonymous SNVs in Yunong 201 and 3114. **(D–F)** Three specific kinds of SNVs, namely, stop–gain, stop–loss, and stop–stop.

As the wheat reference sequence in URGI (Unité de Recherche Génomique Info)^8^ was mapped, SNVs in both Yunong 201 and 3114 were detected. A large number of genetic variations were identified across the transcriptomes of both strains, including non-synonymous, synonymous, stop–gain, stop–loss, and stop–stop SNVs (**Figure 2**). To maintain data credibility, the non-synonymous SNVs that could be simultaneously annotated in reference sequence were analyzed. Non-synonymous SNVs accounted for 49.21% (5904) and 50.32% (6287) in Yunong 201 and 3114 of overall variations occurring in CDS regions, respectively (**Table 1**). A total of 7432 same mutated positions in Yunong 201 and 3114 were also observed, including 3714 synonymous SNVs and 3718 non-synonymous SNVs. Yunong 3114 had 2569 distinct non-synonymous SNVs, and Yunong 201 had 2186 distinct non-synonymous SNVs as compared to reference sequence (**Table 1**). Furthermore, one SNV site may induce both synonymous and non-synonymous mutations that were acted as the multi-SNVs. A total of 22 and 18 multi-SNVs in Yunong 201 and 3114 were recorded, respectively.

**Table 1:** Single nucleotide variants in Yunong 201 and 3114 based on reference sequence.

	Total	ss	nss	Same ss	Same nss	Distinct ss	Distinct nss	Multi-SNVs
Yunong 201	11,997	6093	5904 (49.21%)	3714	3718	2379	2186	22
Yunong 3114	12,495	6208	6287 (50.32%)			2494	2569	18

*ss, short for synonymous substitution; nss, non-synonymous substitution.*

**FIGURE 3:**
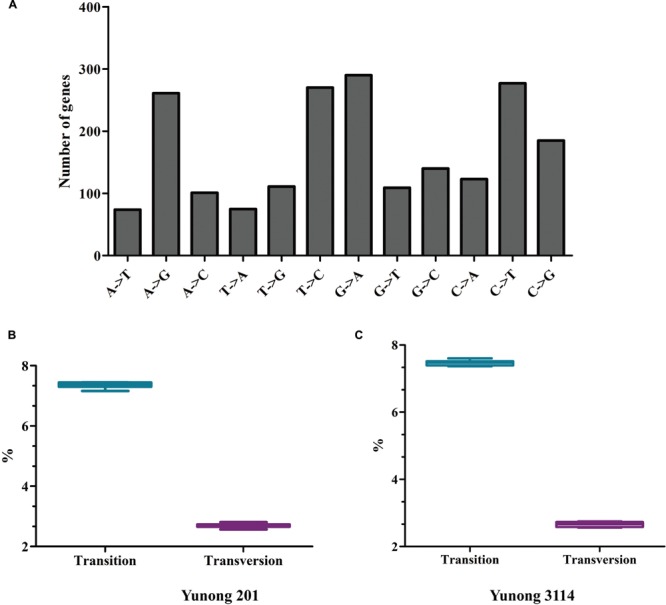
**Patterns of nucleotide substitution for Yunong 201 and 3114 based on reference sequence. (A)** Number of genes with different type of nucleotide substitution. **(B,C)** Percentage (%) of transition and transversion substitutions in Yunong 201 and Yuong 3114. Unpaired *t*-test was used, *P*-value < 0.0001.

Ethyl methanesulfonate preferentially induces G residues alkylation, thereby resulting in many G→A and C→T transitions [CG→TA; (Greene et al., 2003; Till et al., 2007; Tsai et al., 2011; Monson-Miller et al., 2012)]. Thus, in accordance with previous findings, C→T and G→A transitions were the most prevalent patterns of nucleotide substitutions (**Figure 3A**; **Supplementary Figure S1**), and the total GC content of Yunong 201 was higher than that of Yunong 3114. Based on the reference wheat sequence, the transitions were higher than transversions both in Yunong 201 and in 3114 (**Figures 3B,C**). Moreover, the mutations in the first and second codes were significantly more frequent than the third code, both in Yunong 201 and 3114. Our data indicated that the genetic variations were significantly different between the Yunong wheat strains and reference sequence; meanwhile, the difference was smaller between Yunong 201 and 3114, which is mainly because Yunong 3114 was derived from Yunong 201.

### SNVs and Gene Functional Analysis

**FIGURE 4:**
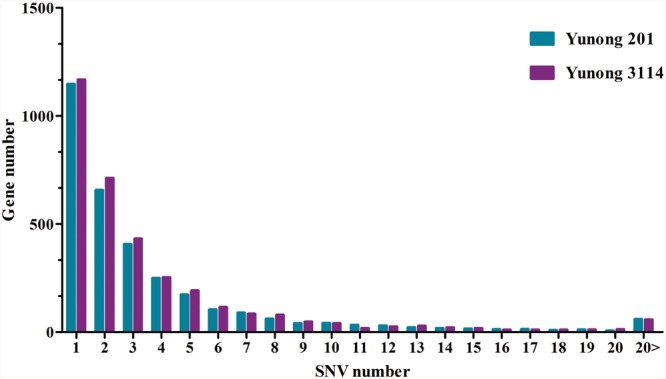
**Relationship between SNV and genes in Yunong 201 and 3114 based on the reference sequence.** The x axis represents the number of SNV mutations on a single gene sequence. The y axis showed number of genes with SNVs. The number of genes with one SNV ranks as the top.

**FIGURE 5:**
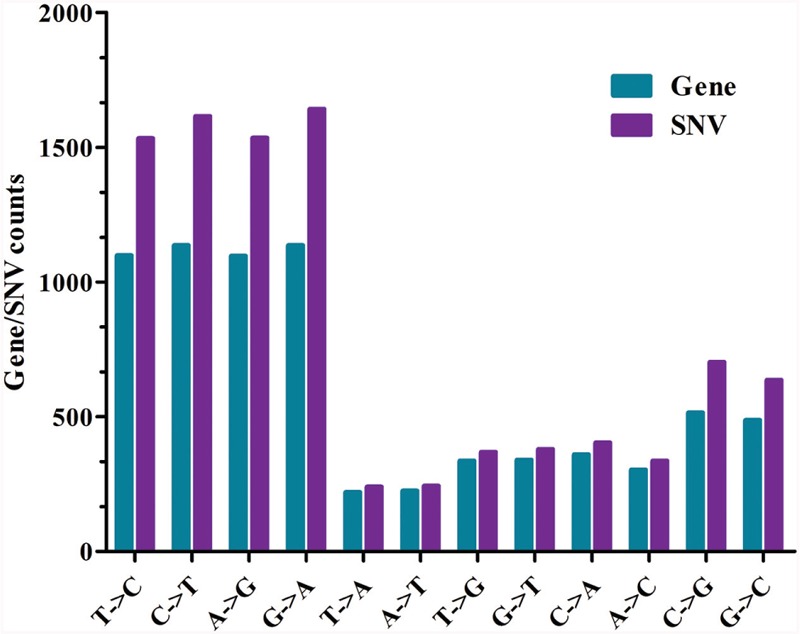
**Mutation analysis in Yunong 3114 and 201.** The x axis indicates the type of mutations; the y axis represents the number of mutant genes or number of SNVs.

**FIGURE 6:**
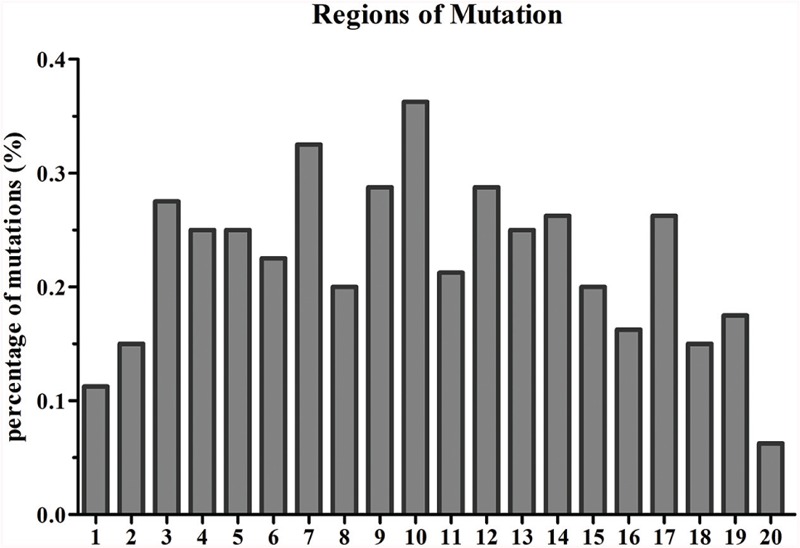
**Single nucleotide variant regions in the gene.** A gene was equally divided into 20 regions, the percentage of mutant in each region was statistically calculated. The y axis showed the percentage of mutant; the x axis represented the gene regions.

Based on the reference sequence, a total of 4021 genes with SNVs were acquired, and most genes have one mutated position (**Figure 4**). A total of 1147 genes in Yunong 201 had one mutant position where 576 genes underwent a non-synonymous mutation (**Figure 4**). Meanwhile, a total of 1167 genes had one SNV positions in Yunong 3114, 564 of which had a non-synonymous mutation. We also detected 4755 non-synonymous SNVs and 1168 genes with non-synonymous mutations by comparing Yunong 3114 with Yuong 201 (**Table 2**). Moreover, genes carrying transistions were more than those undergone to transversions (**Figure 5**). SNVs were distributed in different scaffolds and contigs of the reference genome. EMS-induced mutagenesis produces high mutant rate in plant. Therefore, we also analyzed the mutagenesis frequencies (**Figure 6**). However, the mutation sites were distributed atypically across the transcriptome (**Figure 6**; **Supplementary Figure S2**). The mutant gene numbers in different mutation patterns were determined, as shown in **Supplementary Figure S3**.

**Table 2:** Single nucleotide variants and gene counts between Yunong 3114 and Yunong 201.

	Synonymous	Non-synonymous	Synonymous–non-synonymous
SNV counts	4873	4755	0
gene counts	1127	1168	987

*The last column shows number of genes underwent both synonymous and non-synonymous substitutions.*

**FIGURE 7:**
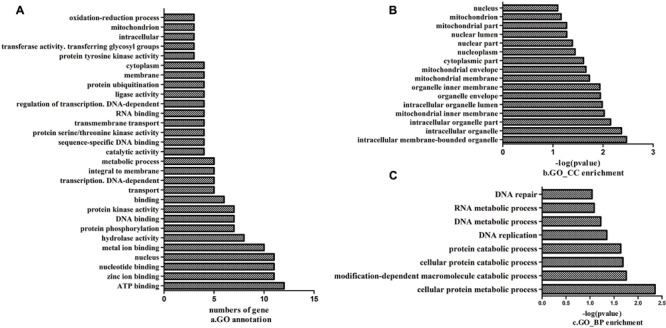
**Function annotation and enrichment for the genes with G→A and/or C→T mutations. (A)** The annotated biological processes; **(B)** The enrichment of cellular component; **(C)** The enrichment of biological process.

**FIGURE 8:**
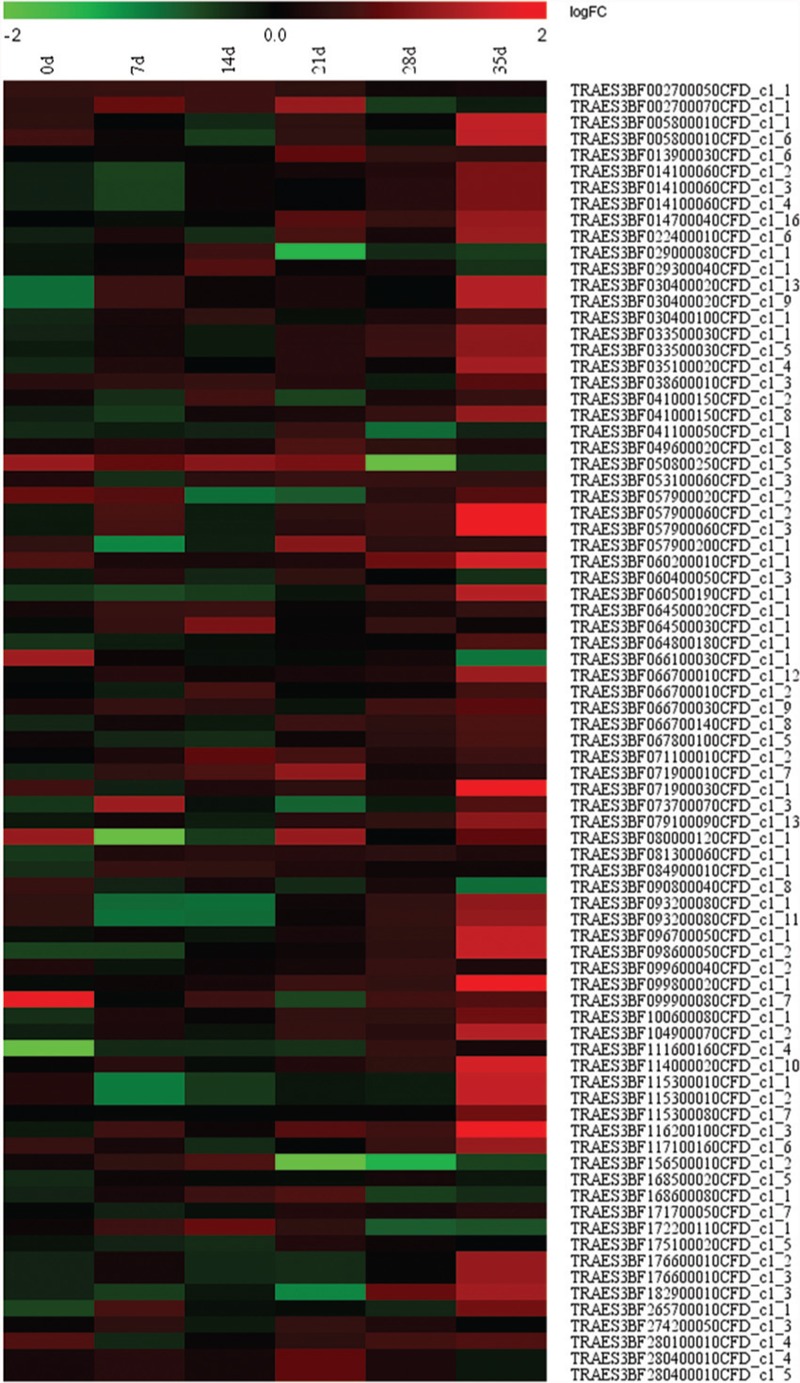
**Preliminary Expression of genes with mutations of G->A and/or C->T between Yunong 201 and Yunong 3114 in different days.** Horizontal axis represent the genes and vertical axis represent the time. The preliminary expression level for a gene in some days was described by the grids, which was calculated by the logFC (FC stands for ‘fold change’). The change of color from green to red represents the expression value from the low to high.

All the mutant genes with G to A or C to T transitions in the mutant strain compared with Yunong 201 were extracted; function annotation and enrichment analyses were performed. These genes were annotated in ATP binding, zinc ion binding, and nucleotide binding (**Figure 7A**); they were also mainly enriched in components, such as intracellular membrane-bound and intracellular organelles (**Figure 7B**); moreover, their functions were enriched in cellular protein metabolic process, modification-dependent macromolecule catabolic process, and cellular protein catabolic process (**Figure 7C**). The preliminary expression levels of these mutant genes were also analyzed by heat map analysis (**Figure 8**). For all the six developmental stages, most of these mutant genes were significantly differentially expressed, which indicated that the SNVs on these genes might be associated with the longer kernel length and higher kernel weight of Yunong 3114.

## Discussion

Next-generation sequencing has been applied in many species, specifically in crop breeding, to explore genomic variations for investigating population evolutionary history, discovering marker-trait linkages, and genetic cause of phenotype variation. EMS-induced mutagenesis is one of the most effective, reliable, powerful, and frequently used technologies in plant. In the present study, Illumina (Solexa) NGS technology was applied to obtain the transcriptomes of bread wheat strain Yunong 201 and its EMS mutant line Yunong 3114. We comprehensively analyzed the SNVs and their impact on genes in Yunong 3114. Our data demonstrated that transcriptomes could also be used for SNV analysis, and a set of SNVs had significant impact on the expression levels of the mutant genes, potentially associated with the superior traits such as the longer kernel length and higher kernel weight of Yunong 3114. However, further validations are necessary.

### Non-synonymous SNVs in Yunong 3114 and 201

Non-synonymous changes directly determine the amino acid change and further affect protein structure, stability, or location. Thus, the identification of non-synonymous sequence variations that can potentially affect gene or protein functionality is the focus of the current study. Stop–gain SNVs may lead to functional consequences due to protein truncation, transcript degradation, and dominant negative influences of protein species (Nagy and Maquat, 1998). The stop–loss non-synonymous SNVs of Yunong 3114 were also significantly less than that of Yunong 201. Current computational methods estimating loss-of-function in genes carrying variants are based on evolutionary conservation and functional redundancy on gene level (Rausell et al., 2014). For all non-synonymous SNVs, [A/G] and [T/C] are the most common base changes both in Yunong 201 and 3114, which agrees with the results of previous studies in legume species (Hand et al., 2008; Deulvot et al., 2010; Gaur et al., 2012; Leonforte et al., 2013). The nucleotide substitution in the first code in Yunong 3114 was significantly more frequent than in Yunong 201. Increased divergence in nucleotide composition shows a corresponding, predictable change in the amino acid compositions of the encoded proteins (Wang et al., 2004). Efficient transcription or mRNA processing is responsible for the high expression of GC-rich genes (Kudla et al., 2006). In early replicating regions, G and C nucleotides are more often misincorporated (Eyre-Walker and Hurst, 2001; Muyle et al., 2011). The distribution of GC content is a remarkable characteristic of genome organization that is often associated with many genomic features, such as meiotic recombination, gene density, and gene length (Mouchiroud et al., 1991; Duret et al., 1995; Duret and Arndt, 2008).

### SNVs Impact on Gene Functionality

Most genes had one to three SNV positions both in Yunong 201 and 3114. The genes that underwent non-synonymous SNVs were significantly involved in ATP binding and metal and zinc ion binding; they specifically regulated protein kinase activity, protein phosphorylation, cellular protein metabolic process, and modification-dependent macromolecular catabolic process. Metal ions are crucial elements in enzymatic reaction events in all photosynthetic organisms, such as cyanobacteria, algae, and plants. Metal ions play important roles in maintaining substrate binding in the active site of metalloenzymes and controlling the redox activity of metalloenzymes in enzymatic reaction (Pospisil, 2014). Some oxidoreductases are also important in the metabolic responses. Protein phosphorylation and protein kinase activity play important roles in the regulation of carbon and nitrogen metabolite production in plants (Huber, 2007). Therefore, SNVs in Yunong 3114 may have impact on these metabolic processes, subsequently influencing the metabolism, energy or nutrient accumulation, and grain growth.

We also analyzed and compared the non-synonymous SNVs of the genes associated with these biological processes in both Yunong 201 and 3114. Some SNVs only occurred in Yunong 3114, and these SNVs potentially implicated the relatively superior trait. If the genomic architectures of the traits studied are highly complex, dozens or even hundreds of causative polymorphisms with minor effects may exist across the genome. For example, SNVs on genes of protein phosphatase 5 (PP5), glycerophosphoryl diester phosphodiesterase (GDPD), dephospho-CoA kinase, and alkylated DNA repair protein alkB homolog 1, cytochrome c oxidase subunit 2, and ABA responsive element binding factor may have impact on wheat gene function, thus may result in changes on biological process associated with grain size. It has been reported that *Arabidopsis* protein phosphatase 5 (AtPP5) performs multi enzymatic activities such as biologically active photoreceptor and protein phosphatase activity (Ryu et al., 2005). The AtPP5 is capable to form a complex with AtHsp90 under heat shock conditions, enhancing thermotolerance in *Arabidopsis* (Park et al., 2012). *Arabidopsis* SNC4 encodes a receptor-like kinase with two predicted extracellular GDPD domains and also plays a role as resistance protein (Bi et al., 2010). Another *Arabidopsis* protein shaven3 also has two tandemly repeated GDPD-like domains which were suggested to be involved in primary cell wall organization (Hayashi et al., 2008). The ABA responsive element binding factor has been found in rice such as OsABF1 (*Oryza sativa* ABA responsive element binding factor 1) gene which is involved in abiotic stress responses and ABA signaling in rice (Amir Hossain et al., 2010), and OREB1 whose phosphorylation plays important roles in regulating signal integration in the complex stress signaling network of plant cells (Hong et al., 2011). SNVs distributed in these genes might trigger alterations on the secondary structure of proteins that possibly impacts the subsequent functions (such as DNA binding and transcriptional activity) of target genes involved in multiple agronomic traits in wheat. The clues obtained from this study could facilitate the further illustration of gene function with the non-synonymous SNVs between Yunong 201 and 3114.

## Conclusion

The current study analyzed the SNVs on transcriptome levels of bread wheat Yunong 201 and its EMS mutagenesis strain Yunong 3114. Our data provided useful information on wheat transcriptome by presenting new resources that could be used in future studies on wheat functional genomics. In addition, detecting new genetic variation for wheat and further information on gene functions that eventually generate new phenotypic variation provide insight into wheat breeding program.

## Author Contributions

FC designed the project. FC, ZZ, and XZ performed RNA-seq experiments. FC wrote the paper. FC, ZD, YY, and DC performed the computational analyses.

## Conflict of Interest Statement

The authors declare that the research was conducted in the absence of any commercial or financial relationships that could be construed as a potential conflict of interest.

## Footnotes

1 http://sourceforge.net/projects/seqclean/

2 https://urgi.versailles.inra.fr/blast/blast.php

3 http://www.ncbi.nlm.nih.gov/

4 ftp://occams.dfci.harvard.edu/pub/bio/tgi/data

5 https://urgi.versailles.inra.fr/blast/blast.php

6 http://www.geneontology.org/

7 http://www.genome.jp/kegg/

8 https://urgi.versailles.inra.fr/

## Supplementary Material

FIGURE S1 **Patterns of nucleotide substitution in the SNVs of Yunong 201 and 3114 based on the reference genome. (A)** Nucleotide substitutions of the SNVs for Yunong 201; **(B)** Nucleotide substitutions of the SNVs for Yunong 3114.

FIGURE S2 **Different SNVs of 18 functional genes between Yunong 201 and 3114.** Boxes with letters show the mutant positions.

FIGURE S3 **Mutant gene numbers in different mutation patterns in both Yunong 201 and 3114**.
