# Supplementary figures and images for: High-Throughput Sequencing Reveals Single Nucleotide Variants in Longer-Kernel Bread Wheat
Source: Front Plant Sci. 2016 Aug 8;7:1193. doi: 10.3389/fpls.2016.01193 (PMC4976665; doi:10.3389/fpls.2016.01193)

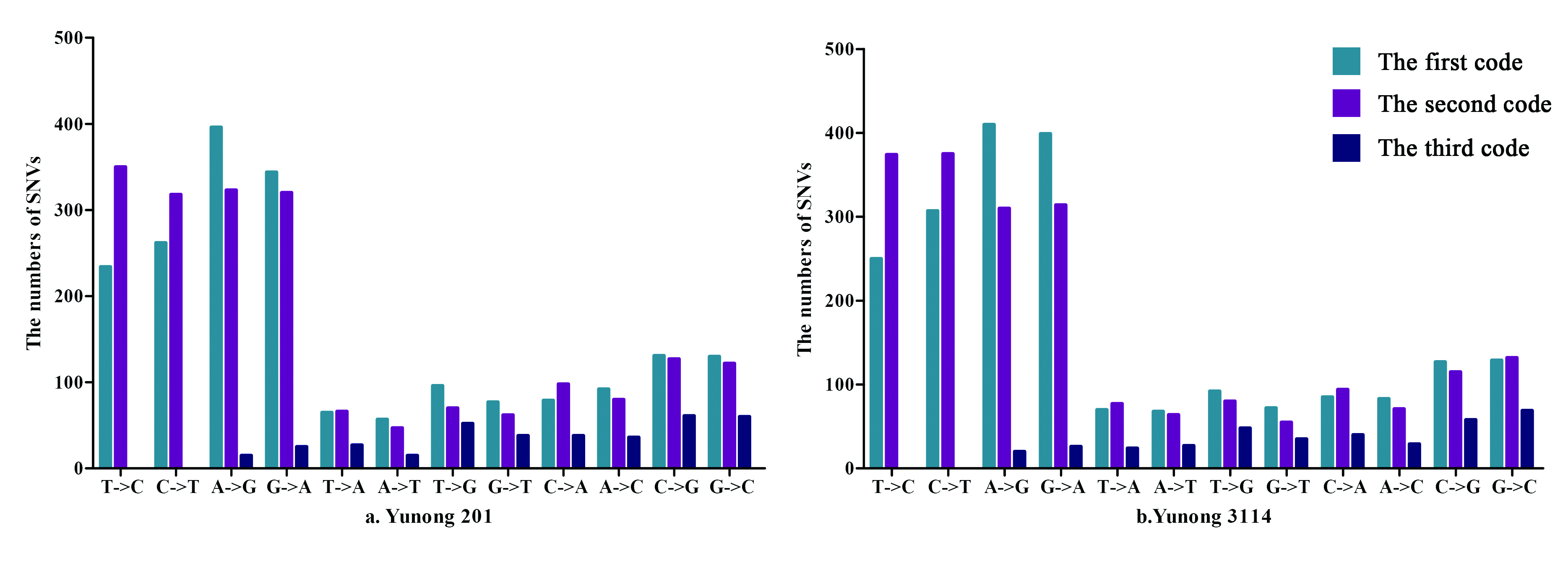

Supplement: FIGURE S1 — Patterns of nucleotide substitution in the SNVs of Yunong 201 and 3114 based on the reference genome. (A) Nucleotide substitutions of the SNVs for Yunong 201; (B) Nucleotide substitutions of the SNVs for Yunong 3114. [file Image_1.JPEG]

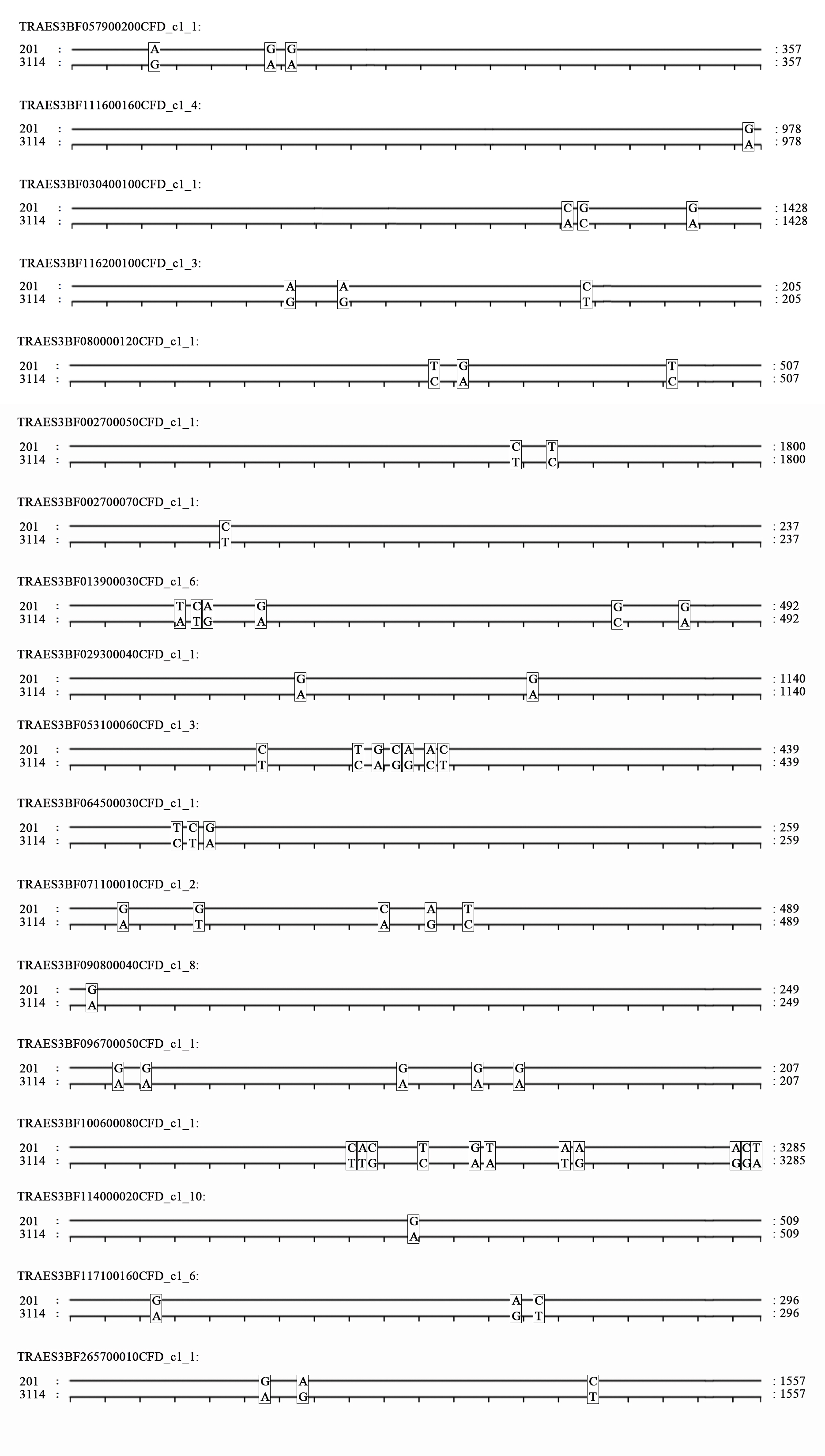

Supplement: FIGURE S2 — Different SNVs of 18 functional genes between Yunong 201 and 3114. Boxes with letters show the mutant positions. [file Image_2.JPEG]

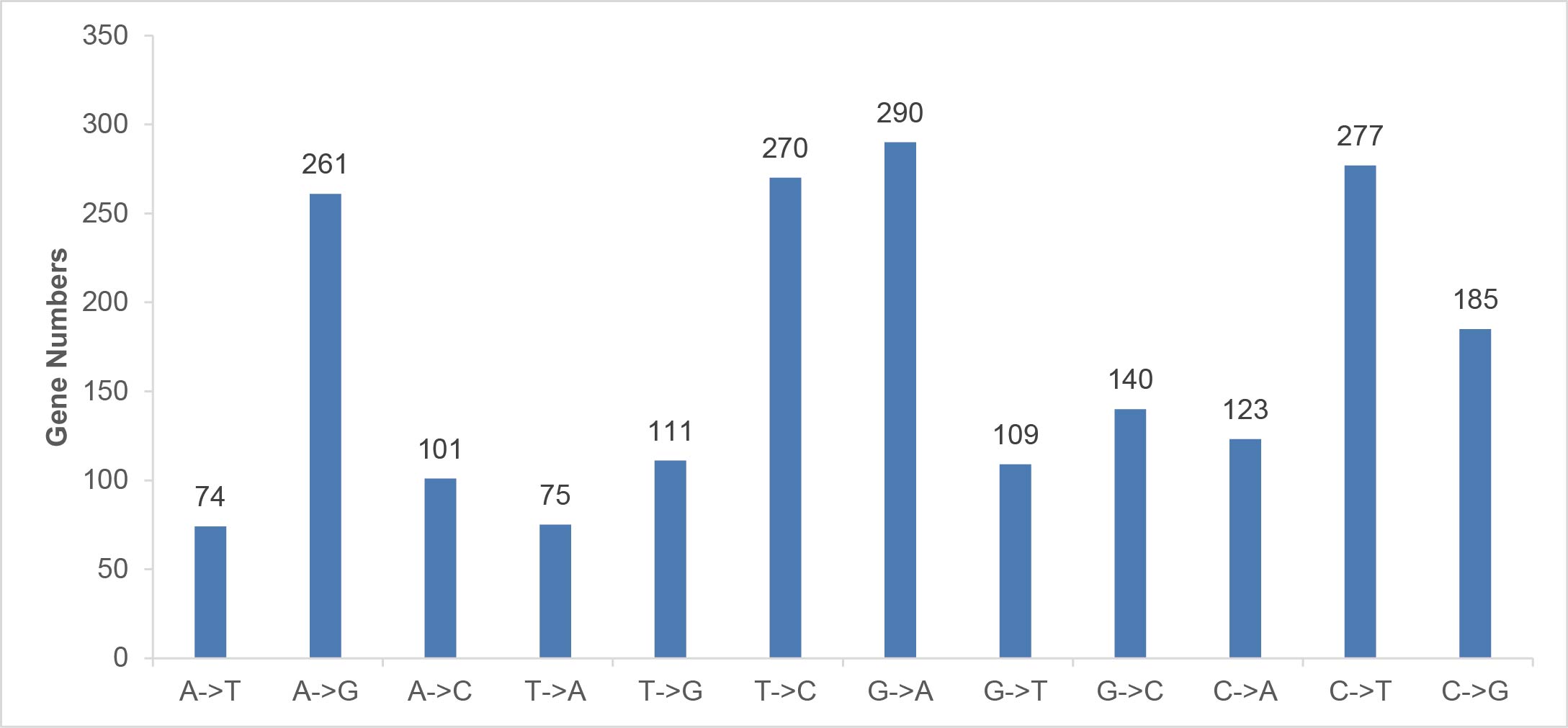

Supplement: FIGURE S3 — Mutant gene numbers in different mutation patterns in both Yunong 201 and 3114. [file Image_3.JPEG]
